# Changing Etiological Patterns and Predictors of In-Hospital Mortality in Decompensated Cirrhosis: A 10-Year Cohort Study Before and After COVID-19

**DOI:** 10.3390/diseases14060203

**Published:** 2026-06-06

**Authors:** Lavinia Alice Bălăceanu, Claudia Georgeta Iacobescu, Ioana Valeria Grigorescu, Ion Daniel Baboi, Marian-Vlad Lapadat, Ion Dina

**Affiliations:** 1 Clinical Department 1—Medical Semiology, “Carol Davila” University of Medicine and Pharmacy, 020021 Bucharest, Romania; alice.balaceanu@umfcd.ro (L.A.B.); ion.dina@umfcd.ro (I.D.); 2 Internal Medicine Department, Clinical and Emergency Hospital “Sf. Ioan”, 042122 Bucharest, Romania; 3 Gastroenterology Department, Clinical and Emergency Hospital “Sf. Ioan”, 042122 Bucharest, Romania; iacobescu_clodi@yahoo.com (C.G.I.); ioana-valeria.grigorescu@rez.umfcd.ro (I.V.G.)

**Keywords:** liver cirrhosis, decompensated cirrhosis, COVID-19, thrombocytopenia, alcohol-related cirrhosis

## Abstract

Background: Decompensated cirrhosis is associated with high in-hospital mortality, influenced by disease severity and underlying etiology. The COVID-19 pandemic may have altered both the etiological spectrum and clinical presentation of hospitalized patients. This study aimed to assess longitudinal changes in etiology and identify predictors of in-hospital mortality over a 10-year period. Methods: We conducted a retrospective cohort study including 812 patients hospitalized with decompensated liver cirrhosis between 2015 and 2025. Patients were grouped into pre-COVID-19 (2015–2019), COVID-19 (2020–2021) and post-COVID-19 (2022–2025) periods. Etiological factors and mortality rates were compared using chi-square tests. Independent predictors were identified through multivariate analysis. A clinical risk score based on Child–Pugh stage, platelet count and age was developed and evaluated using ROC analysis. Results: Alcohol-related cirrhosis increased significantly from 51.2% (pre-COVID-19) to 90.4% (COVID-19) and remained high post-COVID-19 (86.3%) (*p* < 0.001), while HCV decreased from 34.4% to 13.5% and stabilized at 14.8% (*p* < 0.001). HBV showed no significant variation. All-cause in-hospital mortality increased from 19.7% pre-COVID-19 to 42.3% during COVID-19 and remained elevated post-COVID-19 at 34.5% (*p* < 0.001). Independent predictors of all-cause in-hospital mortality included advanced Child–Pugh stage, thrombocytopenia and age above 70 years. The risk score (0–7 points) showed good discrimination (AUC = 0.752), with mortality rates of 2.8%, 24.0% and 45.7% across increasing risk categories. A score <5 had a negative predictive value of 84.3%. Conclusions: A significant etiological shift from HCV to alcohol was observed, accompanied by persistently increased mortality after COVID-19. Thrombocytopenia remains an important predictor of mortality. The proposed score enables simple and effective risk stratification.

## 1. Introduction

Liver cirrhosis represents the end stage of chronic liver disease and remains a major global health burden, with high morbidity and mortality, accounting for every 1 out of 25 deaths worldwide [1]. The most common etiologies of chronic liver disease are related to viral hepatitis, alcohol consumption and non-alcoholic fatty liver disease (NAFLD), also referred to as metabolic dysfunction-associated steatotic liver disease (MASLD) [2,3,4]. The clinical course of cirrhosis is influenced by both disease severity and underlying etiology, which may affect progression, complications and outcomes. The transition from early compensated stages to decompensated cirrhosis is marked by the development of complications, which significantly worsen prognosis [5,6]. Portal hypertension is a key driver of disease progression, leading to severe complications including variceal bleeding—one of the most life-threatening events—as well as hepatic encephalopathy, spontaneous bacterial peritonitis, hepatorenal syndrome and hepatocellular carcinoma, which represent major clinical concerns [7,8,9,10]. Once decompensation occurs, prognosis worsens significantly, with in-hospital mortality rates remaining substantial despite advances in medical management [11]. Over the past decade, the etiological pattern of liver cirrhosis has undergone important changes due to various factors. The progress of medical treatments, particularly the emergence of direct-acting antivirals, has led to a marked decline in hepatitis C virus-related cirrhosis, while alcohol-related liver disease has become a leading cause in many regions, including Romania [12]. Hepatitis B virus (HBV) infection, although better controlled through vaccination and antiviral therapy, continues to contribute to the burden of cirrhosis in certain populations [13]. The COVID-19 pandemic introduced an additional layer of complexity, disrupting healthcare systems worldwide and potentially altering both disease presentation and access to care. Reduced hospital admissions, delayed diagnosis, limited follow-up and changes in alcohol consumption patterns may have influenced the epidemiological profile and severity of cirrhosis at presentation [14]. Emerging evidence suggests that these factors may have contributed to worse clinical outcomes, including increased mortality among hospitalized patients with liver disease. Moreover, cirrhosis is associated with significantly increased all-cause mortality among patients with COVID-19 compared to individuals without underlying liver disease, reflecting the increased vulnerability of this population to severe outcomes [15]. In this context, identifying reliable predictors of in-hospital mortality remains essential for risk stratification and clinical decision-making. Established prognostic tools, such as the Child–Pugh score, are widely used in clinical practice; however, additional readily available clinical parameters may improve predictive accuracy and facilitate early identification of high-risk patients. The present study aimed to evaluate longitudinal changes in the etiological profile of liver cirrhosis over a 10-year period spanning the pre-COVID-19, COVID-19 and post-COVID-19 eras and to assess their association with in-hospital mortality. Additionally, we sought to identify independent predictors of mortality and to develop a simplified clinical risk score based on routinely available parameters.

## 2. Materials and Methods

### 2.1. Study Design and Population

We conducted a retrospective observational cohort study of patients with decompensated liver cirrhosis admitted to a tertiary care hospital over a 10-year period (2015–2025). The initial database included 6801 hospital admissions. To ensure a patient-level analysis, repeated hospitalizations of the same patient were screened and only the first eligible admission per patient was included. After application of inclusion and exclusion criteria, a total of 812 unique patients with complete clinical and laboratory data were included in the final analysis. For temporal analysis, patients were stratified into three predefined periods, including pre-COVID-19 (2015–2019), COVID-19 (2020–2021) and post-COVID-19 (2022–2025), reflecting major epidemiological and healthcare system changes. The COVID-19 period included a smaller number of hospitalizations, reflecting pandemic-related disruptions in healthcare access and admission patterns. The study included both derivation and internal evaluation of a simplified exploratory risk score.

### 2.2. Data Collection

Demographic, clinical, laboratory and etiological data were extracted from electronic medical records. Variables included age, sex and etiology of cirrhosis (alcohol-related liver disease, hepatitis C virus, and hepatitis B virus), Child–Pugh classification, platelet count, and in-hospital mortality. The MELD-Na score was not calculated due to incomplete availability of key laboratory parameters, particularly INR and serum sodium, across the study period. Etiology was assigned based on the predominant causative factor documented in clinical records. In cases of mixed etiology, the dominant clinical cause was determined based on a priority-based clinical assessment considering documented alcohol intake, confirmed viral hepatitis status, and overall clinical judgment recorded in medical charts. Alcohol-related etiology was prioritized in cases of significant and sustained alcohol consumption. Child–Pugh classification was determined using standard clinical criteria, including serum bilirubin, serum albumin, INR, ascites and hepatic encephalopathy, as recorded at hospital admission. MASLD and MetALD were not systematically recorded as separate diagnostic entities in the medical records during the study period and, therefore, could not be analyzed as distinct etiological categories. This rule-based classification approach may introduce a degree of misclassification bias in cases of overlapping etiologies; however, it reflects real-world clinical documentation practices and was applied consistently across the entire cohort.

### 2.3. Outcome Definition

The primary outcome was all-cause in-hospital mortality, defined as death occurring during the same period of hospitalization, regardless of immediate cause.

### 2.4. Statistical Analysis

Categorical variables were expressed as frequencies and percentages, and continuous variables were expressed as means or medians, depending on distribution. Normality of continuous variables was assessed using visual inspection of histograms and Q–Q plots, as well as the Shapiro–Wilk test. Group comparisons across time periods were performed using the chi-square test for categorical variables and ANOVA or Kruskal–Wallis tests for continuous variables, as appropriate. Trend analysis over time was assessed using chi-square tests and Spearman correlation was applied where appropriate. Missing data were minimal (<5% for all variables) and were handled using complete-case analysis. No imputation methods were applied. Outliers were assessed using boxplot visualization and clinical plausibility checks; no values were excluded unless attributable to clearly identifiable data entry errors. Independent predictors of in-hospital mortality were identified using multivariate logistic regression. Cox proportional hazards regression was additionally performed as an exploratory sensitivity analysis and hazard ratios (HRs) were reported with 95% confidence intervals (CIs), acknowledging the absence of post-discharge follow-up data. A clinical risk score was developed based on variables independently associated with mortality (Child–Pugh class, platelet count, and age). The score was evaluated for discrimination using receiver operating characteristic (ROC) curve analysis, with area under the curve (AUC) reported. Sensitivity, specificity and negative predictive value (NPV) were calculated for clinically relevant thresholds. NPV was calculated both for the binary classification threshold (≥5 points) and the predefined low-risk category (0–2 points), reflecting complementary clinical use cases. All statistical analyses were performed using IBM SPSS Statistics version 27.0 (IBM Corp., Armonk, NY, USA). No external packages or additional programming languages were used. A two-sided *p*-value < 0.05 was considered statistically significant. The SPSS modules used are listed in the table below.
**Analysis Type****SPSS Procedure**Tests of normalityExplore procedure (Shapiro–Wilk test, Q–Q plots, histograms).Categorical comparisonsCrosstabs → Chi-square test (Pearson), with Fisher’s exact test when expected cell counts were <5Continuous comparisonsOne-way ANOVA (for normally distributed variables); Kruskal–Wallis H test (for non-parametric distributions)Trend analysisChi-square test for trend (Cochran–Armitage); Spearman’s rank correlation (Spearman’s rho)Multivariable logistic regressionBinary logistic regression (enter method); odds ratios (OR) with 95% confidence intervals (CI) reportedCox regressionSurvival analysis (Cox regression); hazard ratios (HR) with 95% confidence intervals (CI); proportional hazards assumption assessed using Schoenfeld residualROCROC curve analysis; area under the curve (AUC) with 95% confidence intervals (CI).MulticollinearityLinear regression; collinearity diagnostics (VIF); VIF < 5 for all included predictors

### 2.5. Ethical Considerations

The study was conducted in accordance with the Declaration of Helsinki and approved by the Institutional Ethics Committee of St. John Emergency Hospital, Approval No. 4351, date 17 March 2026, obtained prior to manuscript submission. In accordance with the institutional hospital policy, all patients sign a general informed consent form at the time of hospital admission, which includes consent for the use of anonymized clinical data for research and academic purposes. For patients who presented with altered mental status or hepatic encephalopathy, which impaired their decision-making capacity, informed consent for hospitalization and data use was obtained from a legal representative or family member in accordance with institutional procedures and national regulations.

### 2.6. Clinical Risk Score Development

A simplified clinical risk score was developed based on variables independently associated with in-hospital mortality in multivariate logistic regression analysis. The score incorporated Child–Pugh class, platelet count and age. Each variable was assigned weighted points according to the strength of association observed in the regression model. Due to the retrospective nature of the study and the incomplete availability of laboratory parameters required for MELD-Na calculation, MELD-based comparison was not performed. 

## 3. Results

### 3.1. Baseline Characteristics of the Study Population

**Figure 1:**
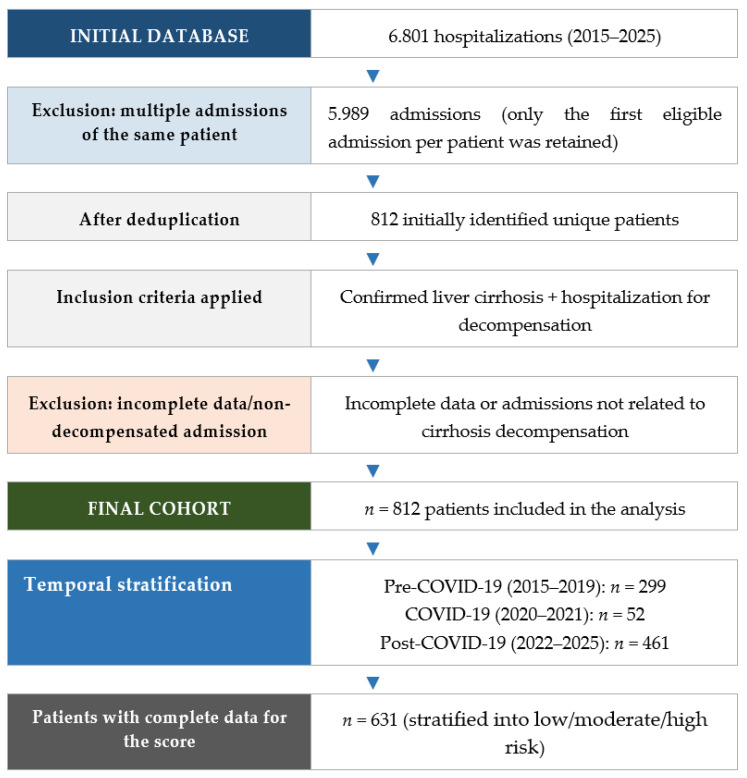
Complete flow diagram (CONSORT-adapted format).

The study included 812 patients with liver cirrhosis, stratified into three temporal periods: pre-COVID-19 (2015–2019), COVID-19 (2020–2021), and post-COVID-19 (2022–2025). A complete flow diagram is depicted in Figure 1. The overall cohort was predominantly male (70–75%), with a mean age of approximately 60 years. Alcohol was the leading etiological factor (60.5%), followed by hepatitis C virus (HCV) (21.9%) and hepatitis B virus (HBV) infection (8.6%). The overall in-hospital mortality rate was 29.6%. The median platelet count was 122 × 10^3^/µL. Detailed baseline characteristics are presented in Table 1.

Table 1 has been expanded to include the detailed distribution across periods, together with the corresponding measures of dispersion, as shown in Table 2 and Table 3. A footnote has been added to clarify the methodology used for calculating the aggregated rate.

### 3.2. Etiological Distribution of Liver Cirrhosis

**Figure 2:**
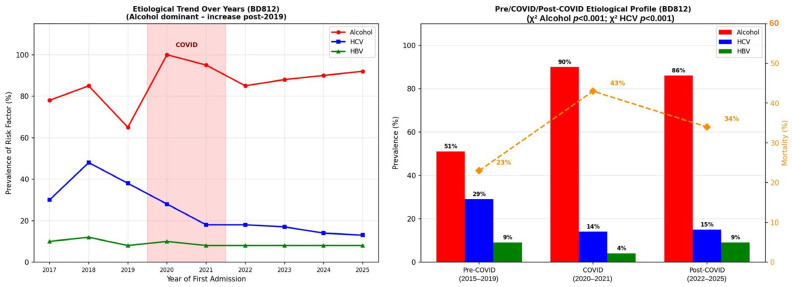
(**Left**): Annual etiological trends (risk factors, %). (**Right**): Comparison of pre-COVID-19, COVID-19 and post-COVID-19 etiological profile and mortality. The red shade represents the temporal limits of the Covid-19 pandemic in Romania.

Alcohol-related cirrhosis was the predominant etiology throughout the study period, with a marked increase over time. A significant shift in etiological distribution was observed across the three predefined periods (pre-COVID-19, COVID-19 and post-COVID-19). Alcohol-related cirrhosis increased from 51.2% in the pre-COVID-19 period to 90.4% during COVID-19, followed by a slight decrease to 86.3% in the post-COVID-19 period (χ^2^ = 123.6, *p* < 0.001). In contrast, HCV-related cirrhosis showed a marked reduction from 34.4% in the pre-COVID-19 period to 13.5% during COVID-19, and remained relatively stable at 14.8% post-COVID-19 (χ^2^ = 43.4, *p* < 0.001). HBV-related cirrhosis did not show significant variation over time (χ^2^ = 1.65, *p* = 0.439), indicating a stable contribution across all periods. Overall, these findings demonstrate a significant and persistent shift in the etiological profile of liver cirrhosis over the 10-year study period, characterized by the increasing dominance of alcohol-related disease and a sustained reduction in HCV-related cases (Table 4, Figure 2).

Figure 2 provides a graphical representation of the etiological trends reported in Table 4, showing two complementary perspectives: the annual evolution over the 2017–2025 period (left panel) and the comparative distribution across the pre-COVID-19, COVID-19 and post-COVID-19 periods with overlaid mortality rates (right panel). The two panels are complementary: the first illustrates when changes occurred, while the second highlights their magnitude and association with mortality.

Left panel—annual trend (study cohort, 2017–2025; *n* = 812):

The time series shows a progressive, non-acute change in etiological structure, with a clear inflection point around 2019–2020. Alcohol-related cirrhosis increases sharply from ~51% to >90% during 2020–2021 and remains elevated thereafter (83–90%). In contrast, HCV decreases from ~30–40% to ~13–15% and remains stable at this lower level. HBV shows no consistent temporal trend, remaining relatively stable across the study period.

Right panel—comparison between periods with mortality overlay:

Across the three periods, alcohol increases (51% → 90% → 86%), HCV decreases (34% → 14% → 15%) and HBV remains stable. Mortality followed a similar pattern, increasing during the COVID-19 period (19.7% → 42.3%) and partially decreasing post-COVID-19 (34.5%) while remaining above the pre-COVID-19 level. Overall, the figure confirms a persistent shift in etiological structure, with a transition from viral hepatitis to alcohol-related cirrhosis, temporally associated with increased mortality.

### 3.3. In-Hospital Mortality

In-hospital mortality rates varied significantly across the three study periods (Table 5). A marked increase was observed during the COVID-19 period, followed by a partial decline post-COVID-19; however, mortality remained significantly higher compared to the pre-COVID-19 baseline. Specifically, in-hospital mortality increased from 19.7% in the pre-COVID-19 period to 42.3% during COVID-19 and decreased to 34.5% in the post-COVID-19 period (χ^2^ = 18.6, *p* < 0.001).

### 3.4. Temporal Changes in Etiological and Clinical Profile

The COVID-19 pandemic (2020–2021) represented a major disruption to the healthcare system, with measurable effects on the epidemiological profile of liver cirrhosis. This comparative analysis included three periods: pre-COVID-19 (*n* = 299), COVID-19 (*n* = 52) and post-COVID-19 (*n* = 461). Alcohol-related cirrhosis showed a significant and sustained increase (51.2% → 90.4% → 86.3%; χ^2^ = 123.6, *p* < 0.001), while HCV-related cirrhosis decreased significantly and remained low (34.4% → 13.5% → 14.8%; χ^2^ = 43.4, *p* < 0.001). HBV-related cirrhosis remained stable (*p* = 0.439). Mortality increased significantly during the COVID-19 period and remained elevated post-COVID-19 (19.7% → 42.3% → 34.5%; χ^2^ = 18.6, *p* < 0.001). Interestingly, the proportion of patients with Child–Pugh class C decreased over time (51.8% → 48.1% → 35.8%; χ^2^ = 18.2, *p* < 0.001), suggesting a paradoxical trend toward a less severe clinical profile despite increased mortality. Despite the observed increase in in-hospital mortality over time, the proportion of patients classified as Child–Pugh class C showed a decrease across study periods. This apparent discrepancy is based on unadjusted descriptive comparisons and may reflect changes in admission patterns, case selection, and healthcare access over time rather than a true reduction in disease severity. Platelet count and mean age remained stable across periods (Kruskal–Wallis test and ANOVA, respectively; both not significant). A comprehensive comparison of etiological and clinical variables across the three study periods is presented in Table 6.

### 3.5. Clinical Predictors of Mortality

Child–Pugh class showed the strongest association with in-hospital mortality, with a progressive increase from Child A (1.2%) to Child B (9.0%) and Child C (46.7%) (*p* < 0.001). Thrombocytopenia was also significantly associated with increased mortality, particularly in patients with platelet counts <100.000/µL (36.5%). Age ≥ 70 years was identified as an independent but weaker predictor of mortality (HR = 1.19).

### 3.6. Development of the Clinical Risk Score

A simplified exploratory clinical risk score was developed based on variables independently associated with in-hospital mortality in multivariate logistic regression analysis. The final model included three predictors: Child–Pugh classification, platelet count and age (Table 7). Points were assigned according to the strength of association observed in the multivariate model. The total score ranged from 0 to 7 points, with higher values indicating increased risk of in-hospital mortality. Patients were stratified into three predefined risk categories: low risk (0–2 points), intermediate risk (3–4 points) and high risk (5–7 points). Observed in-hospital mortality increased progressively across risk categories, from 2.8% in the low-risk group to 24.0% in the intermediate-risk group and 45.7% in the high-risk group (χ^2^ = 86.9, *p* < 0.001). Child–Pugh class contributed most strongly to the score, followed by platelet count and age. This score was evaluated internally within the study cohort as an exploratory tool and requires external validation before clinical application.

### 3.7. Performance of the Clinical Risk Score

The performance of the simplified exploratory clinical risk score was assessed within the study cohort (*n* = 631). Patients were stratified into three predefined risk categories based on total score values: low risk (0–2 points), intermediate risk (3–4 points) and high risk (5–7 points). A clear and statistically significant stepwise increase in in-hospital mortality was observed across the three groups, rising from 2.8% in the low-risk category to 24.0% in the intermediate-risk category and 45.7% in the high-risk category (χ^2^ = 86.9, *p* < 0.001) (Table 8). This progressive gradient supports the clinical discriminatory ability of the proposed scoring system in stratifying patients according to mortality risk. The model demonstrated acceptable discriminatory performance, with an area under the receiver operating characteristic (ROC) curve of 0.752, indicating good ability to distinguish between survivors and non-survivors within the studied cohort. In addition, the predefined low-risk category (score 0–2) showed a negative predictive value of 97.2% for in-hospital mortality. At the optimal cutoff value of ≥5 points, the score achieved a sensitivity of 68.3% and a specificity of 67.6%, suggesting a balanced performance for identifying high-risk patients while maintaining moderate false-positive rates. The negative predictive value of the binary classification at the predefined cutoff (≥5 points) was 84.3%. It is important to emphasize that this predictive model was derived and evaluated in a single-center retrospective cohort, without external validation. Furthermore, no internal resampling techniques such as bootstrapping or cross-validation were applied, which may limit the robustness of the reported performance metrics and increase the risk of model optimism. As such, the observed discriminative ability should be interpreted as an internal, exploratory estimate rather than definitive clinical validation. Additionally, the number of outcome events in the low-risk group was limited (*n* = 4 deaths), which may reduce the statistical stability of subgroup-specific estimates, particularly the negative predictive value. This imbalance in event distribution across risk categories may introduce uncertainty in the lower end of the risk spectrum and should be considered when interpreting the clinical applicability of the score. Overall, these findings suggest that the proposed score may serve as a simple and clinically applicable tool for early risk stratification in patients with decompensated cirrhosis. However, given the methodological limitations of the study, including its retrospective design, single-center setting, absence of external validation and limited event numbers in the low-risk category, the score should be considered hypothesis-generating and requires prospective validation in larger independent cohorts before clinical implementation.

The initial presentation of the diagnostic performance metrics was incomplete; therefore, the full performance parameters for the predefined threshold of ≥5 points (high-risk category) have now been calculated in the complete analyzable cohort (*n* = 631 patients with complete scoring data), and these results are presented in Table 9.

The analysis included 631 patients with complete data (Child–Pugh score, platelet count, and age). The optimal threshold of ≥5 points provides a balance between sensitivity and specificity. The negative predictive value in the low-risk category (97.2%) confirms its clinical utility in ruling out high mortality risk.

**Figure 3:**
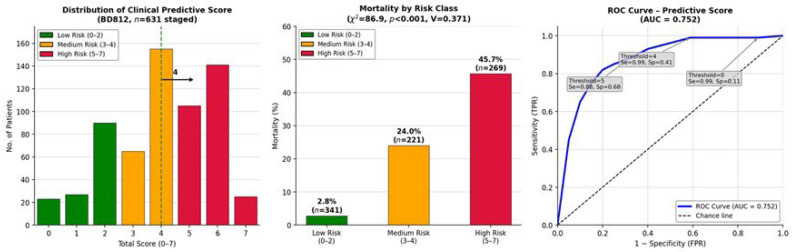
Performance of the simplified exploratory clinical risk score. The **left** panel shows the distribution of total score values (0–7) among the study cohort. The **middle** panel illustrates observed in-hospital mortality across risk categories (low, intermediate and high risk). The **right** panel shows the receiver operating characteristic (ROC) curve for the score (AUC = 0.752). The blue dotted line from the left is visually explained as being the demarcation of a score of 4; while the blue line in the right panel is the actual ROC curve.

The distribution of the predictive score, associated mortality across risk categories and ROC curve analysis are illustrated in Figure 3.

Figure 3 integrates three complementary graphical representations of the predictive score performance. The left panel shows the distribution of the total score among the 631 stratified patients, with a non-uniform, right-skewed pattern and the highest frequencies at scores four and five, consistent with a predominance of advanced disease. Dashed vertical lines indicate thresholds between risk categories (two and four points), corresponding to low-(*n* = 141), intermediate-(*n* = 221) and high-risk (*n* = 269) groups. The central panel illustrates mortality across risk categories, demonstrating a clear stepwise increase (2.8%, 24.0%, and 45.7%; χ^2^ = 86.9, *p* < 0.001), supporting the discriminatory capacity of the score. The right panel presents the ROC curve, with an AUC of 0.752, indicating a good discriminatory ability for in-hospital mortality prediction (AUC = 0.752). A threshold of five points provides the optimal balance between sensitivity (0.68) and specificity (0.66), while lower thresholds increase sensitivity at the expense of specificity. Overall, the figure confirms consistent score performance, combining a realistic distribution, a well-defined mortality gradient and good discriminative ability with a negative predictive value of 84.3%, supporting its utility in ruling out high-risk patients.

## 4. Discussions

Liver cirrhosis, particularly in its decompensated stage, remains associated with high short-term mortality, especially during acute hospital admissions [16,17]. This 10-year cohort study demonstrates a significant shift in the etiological profile of decompensated cirrhosis, with a marked increase in alcohol-related disease and a concomitant decline in HCV-related cirrhosis, results that are consistent with data from the literature [1,18]. These observations reflect broader epidemiological changes described in the recent literature over the past decade [19]. These changes are largely attributed to the impact of direct-acting antiviral (DAA) therapies, which have substantially reduced the burden of HCV-related liver disease [20,21]. In contrast, alcohol-related liver disease has emerged as the dominant etiology throughout the study period, with a further increase observed during and after the COVID-19 pandemic [12,22,23]. This trend is consistent with reports from multiple regions, suggesting that increased alcohol consumption, psychosocial stress, and reduced access to healthcare during the pandemic contributed to disease progression and hospital admissions and may reflect a potential sustained effect of delayed diagnosis and healthcare disruption during the pandemic period. Post-COVID-19 in-hospital mortality remained significantly higher compared to the pre-COVID-19 period (34.5% vs. 19.7%; χ^2^ = 18.6, *p* < 0.001), suggesting a sustained impact of delayed diagnosis, reduced healthcare access and disease progression during the pandemic [14,24,25,26]. Portal hypertension is a key pathophysiological driver of decompensation in cirrhosis and may contribute to complications such as variceal bleeding and hepatorenal syndrome, although these were not specifically analyzed in this cohort. In addition, portal vein thrombosis or extensive thrombosis of the portal venous system may further aggravate portal hemodynamics and, in severe cases, result in intestinal ischemia [27]. Interestingly, despite increased mortality, the proportion of patients classified as Child–Pugh C decreased over time, suggesting changes in admission patterns, patient selection or healthcare access during the pandemic. This finding may reflect differences in admission patterns, patient selection and healthcare access during the study periods rather than a true change in disease severity. Among the prognostic factors, Child–Pugh class remained the strongest predictor of in-hospital mortality, followed by thrombocytopenia and age ≥ 70 years. These findings are consistent with established prognostic models in liver cirrhosis [28,29]. Based on these variables, we developed and internally evaluated a simplified exploratory clinical score that demonstrated good discriminatory performance for in-hospital mortality and may facilitate early risk stratification in clinical practice. Although MELD-Na is considered a more objective prognostic score in cirrhosis, it could not be applied in this study due to missing key laboratory variables in a substantial proportion of patients, particularly in the early study period. Overall, our findings highlight a dual transition in cirrhosis epidemiology and outcomes, characterized by a decline in viral hepatitis-related disease and a sustained increase in alcohol-related liver disease, compounded by the indirect effects of the COVID-19 pandemic.

This study has several limitations that should be acknowledged. First, its retrospective single-center design introduces inherent limitations, including potential selection bias, unmeasured confounding and reduced generalizability to other populations and healthcare settings. Second, important clinical data regarding complications of decompensated cirrhosis—such as variceal bleeding, ascites severity, hepatic encephalopathy, spontaneous bacterial peritonitis, hepatorenal syndrome, acute-on-chronic liver failure and intensive care unit admission—were not systematically available for the entire cohort, limiting the ability to perform more detailed adjusted analyses of disease severity and outcomes. Third, several relevant clinical, metabolic and socioeconomic variables, including body mass index, type 2 diabetes, socioeconomic status, duration of liver disease, medication history and detailed liver biochemistry parameters, were incompletely recorded across the study period and therefore could not be included in the analysis. In addition, treatment-related information, such as antiviral therapy, alcohol cessation interventions and other management strategies, was not consistently available, which may have influenced observed outcomes. Moreover, cause-specific mortality could not be reliably assessed due to inconsistent retrospective documentation, and therefore, only all-cause in-hospital mortality was analyzed. Finally, the proposed simplified prognostic score should be considered exploratory and hypothesis-generating, as it was internally derived and has not yet been externally validated. External validation in independent prospective cohorts is required before any clinical application can be considered. Overall, these findings highlight a temporal transition in cirrhosis epidemiology and outcomes, characterized by a decline in viral hepatitis-related disease and a sustained increase in alcohol-related liver disease, potentially influenced by the indirect effects of the COVID-19 pandemic.

## 5. Conclusions

This 10-year cohort study highlights a significant shift in the etiological profile of decompensated cirrhosis, with a sustained increase in alcohol-related disease and a marked decline in HCV-related cirrhosis. The COVID-19 pandemic was associated with persistently higher in-hospital mortality, likely reflecting delayed presentation and disease progression. Thrombocytopenia and advanced liver dysfunction were associated with adverse in-hospital outcomes, although the absence of systematically collected data regarding specific decompensating complications limits further mechanistic interpretation.

## Figures and Tables

**Table 1:** Baseline characteristics of the study population (*n* = 812).

Characteristic	Value
Patients (*n*)	812
Age (years)	60
Male sex (%)	70–75
Alcohol (%)	60.5
HCV (%)	21.9
HBV(%)	8.6
In-hospital mortality (%)	29.6
Platelets (×10^3^/µL)	122

**Table 2:** Distribution of patients and in-hospital mortality across the study periods.

Characteristic	Pre-COVID-19 (2015–2019)	COVID-19 (2020–2021)	Post-COVID-19 (2022–2025)	Total (*n* = 812)
Patient number (*n*)	299	52	461	812
Deceased	59	22	159	240
In-hospital mortality (%)	19.7%	42.3%	34.5%	29.6% *
Male sex (%)	203 (67.9%)	40 (76.9%)	357 (77.4%)	600 (73.9%)
Mean age (years)	60.9	58.4	59.3	59.7
Alcohol (%)	153 (51.2%)	47 (90.4%)	398 (86.3%)	491 (60.5%)
HCV(%)	103 (34.4%)	7 (13.5%)	68 (14.8%)	178 (21.9%)
HBV(%)	26 (8.7%)	2 (3.8%)	42 (9.1%)	70 (8.6%)
Child A (%)	39 (13.0%)	3 (5.8%)	43 (9.3%)	85 (10.5%)
Child B (%)	75 (25.1%)	10 (19.2%)	116 (25.2%)	201 (24.8%)
Child C	107 (35.8%)	35 (67.3%)	203 (44.0%)	345 (42.5%)
Median platelet count (×10^3^/µL)	131	90	122	122

* The overall mortality of 29.6% = 240/812 was calculated as an aggregated rate (total deaths/total patients), rather than as the arithmetic mean of the period-specific rates. The unequal distribution of patients (*n* = 299:52:461) explains the discrepancy compared with the simple mean of the three rates (32.2%).

**Table 3:** Inter-group statistical significance.

Variable	Pre-COVID-19 (*n* = 299)	COVID (*n* = 52)	Post-COVID-19 (*n* = 461)	Test	*p*-Value
Males sex (%)	67.9%	76.9%	77.4%	χ^2^	0.031 *
Mean age (years)	60.9 ± 12.3	58.4 ± 11.7	59.3 ± 11.5	ANOVA	0.412 NS
Alcohol (%)	51.2%	90.4%	86.3%	χ^2^ = 123.6	<0.001 ***
HCV(%)	34.4%	13.5%	14.8%	χ^2^ = 43.4	<0.001 ***
HBV(%)	8.7%	3.8%	9.1%	χ^2^ = 1.65	0.439 NS
Child C	35.8%	67.3%	44.0%	χ^2^ = 18.2	<0.001 ***
Median platelet count (×10^3^/µL)	131	90	122	KW	0.003 **
Mortality (%)	19.7%	42.3%	34.5%	χ^2^ = 18.6	<0.001 ***

Legend: NS = not statistically significant (*p* > 0.05); * *p* < 0.05; ** *p* < 0.01; *** *p* < 0.001. ANOVA = one-way analysis of variance; KW = Kruskal–Wallis test; χ^2^ = Pearson’s chi-square test.

**Table 4:** Etiological trends across time periods.

Risk Factor	Pre-COVID (2015–2019)	COVID (2020–2021)	Post-COVID (2022–2025)	Statistical Analysis
Alcohol-related cirrhosis	51.2%	90.4%	86.3%	χ^2^ = 123.6 *p* < 0.001
HCV-related cirrhosis	34.4%	13.5%	14.8%	χ^2^ = 43.4 *p* < 0.001
HBV-related cirrhosis	8.7%	3.8%	9.1%	χ^2^ = 1.65 *p* = 0.439
All-cause in-hospital mortality	19.7%	42.3%	34.5%	χ^2^ = 18.6 *p* < 0.001

**Table 5:** In-hospital mortality across study periods.

Period	Mortality (%)
Pre-COVID	19.7
COVID	42.3
Post-COVID	34.5
χ^2^	18.6
*p*-value	<0.001

**Table 6:** Comparison of the etiological and clinical profile: Pre-COVID-19 vs. COVID-19 vs. Post-COVID-19.

Indicator	Pre-COVID-19 (*n* = 299)	COVID-19 (*n* = 52)	Post-COVID-19 (*n* = 461)	Significance and Impact
Alcohol (%)	51.2%	90.4%	86.3%	χ^2^ = 123.6, *p* < 0.001
HCV (%)	34.4%	13.5%	14.8%	χ^2^ = 43.4, *p* < 0.001
HBV (%)	8.7(%)	3.8%	9.1%	χ^2^ = 1.65, *p* = 0.439
Mortality (%)	19.7%	42.3%	34.5%	χ^2^ = 18.6, *p* < 0.001
Child C (%)	51.8%	48.1%	35.8%	χ^2^ = 18.2, *p* < 0.001
Median platelet count (×10^3^/µL)	122.000	118.000	124.000	Kruskal–Wallis, NS
Mean age (years)	59.1	61.2	59.9	ANOVA, NS

**Table 7:** Components and point allocation of the clinical mortality risk score.

Component	Criteria	Score	Observed Mortality
Child–Pugh stage	Child A	0 points	1.2%
Child–Pugh stage	Child B	2 points	9.0%
Child–Pugh stage	Child C	4 points	46.7%
Platelet count	<100.000/μL	2 points	36.5%
Platelet count	100.000–150.000/μL	1 point	26.6%
Platelet count	>150.000/μL	0 points	25.7%
Age	≥70 years	1 point	HR = 1.19
Age	<70 years	0 points	Reference category

**Table 8:** Predictive score performance by risk class (*n* = 631 stratified patients).

Risk Class	Score	N Patients	N Deceased	Mortality	Sensibility	Specificity	NPV
Low risk	0–2	141	4	2.8%	-	-	84.3%
Intermediate risk	3–4	221	53	24.0%	-	-	-
High risk	5–7	269	123	45.7%	68.3%	67.6%	-

**Table 9:** Complete performance of the risk score at the predefined threshold (≥5 points).

	Deceased	Survived
Score ≥5 (High risk = positive test)	TP * = 123	FP ** = 146
Score < 5 (Low/moderate risk = negative test result)	FN *** = 57	TN **** = 305
Metric	Value	Formula
Sensitivity	68.3%	TP/(TP + FN) = 123/180
Specificity	67.6%	TN/(TN + FP) = 305/451
PPV	45.7%	TP/(TP + FP) = 123/269
NPV	84.3%	TN/(TN + FN) = 305/362
AUC (ROC curve)	0.752	-
NPV (score 0–2)	97.2%	137/141

Legend: * TP = true positive; ** FP = false positive; *** FN = false negative; **** TN = true negative.

## Data Availability

The raw data supporting the conclusions of this article will be made available by the authors on request due to privacy concerns.

## Abbreviations

The following abbreviations are used in this manuscript:

HCV	hepatitis C virus
HBV	hepatitis B virus
NPV	negative predictive value
PPV	positive predictive value
ROC	receiver operating characteristic
AUC	area under the curve
NAFLD	non-alcoholic fatty liver disease
MASLD	metabolic dysfunction-associated steatotic liver disease
MetALD	metabolic dysfunction-associated alcohol-related liver disease
